# Aetiopathogenesis of infantile epileptic spasms syndrome and mechanisms of action of adrenocorticotrophin hormone/corticosteroids in children: A scoping review

**DOI:** 10.1111/dmcn.16273

**Published:** 2025-02-28

**Authors:** Emily A. Innes, Velda X. Han, Shrujna Patel, Michelle A. Farrar, Deepak Gill, Shekeeb S. Mohammad, Russell C. Dale

**Affiliations:** ^1^ Kids Neuroscience Centre, The Children's Hospital at Westmead University of Sydney NSW Australia; ^2^ Sydney Medical School The University of Notre Dame Australia Sydney NSW Australia; ^3^ Khoo Teck Puat‐National University Children's Medical Institute National University Health System Singapore; ^4^ Yong Loo Lin School of Medicine National University Singapore Singapore; ^5^ Sydney Medical School, Faculty of Medicine and Health University of Sydney Sydney NSW Australia; ^6^ Neurology Department Sydney Children’ Hospital at Randwick Sydney NSW Australia; ^7^ Discipline of Paediatrics, School of Women's and Children's Health, UNSW Medicine The University of New South Wales NSW Australia; ^8^ TY Nelson Department of Neurology and Neurosurgery The Children's Hospital at Westmead Sydney NSW Australia

## Abstract

**Aim:**

To review the aetiopathogenesis of infantile epileptic spasms syndrome (IESS) and mechanisms of action of adrenocorticotrophin hormone (ACTH)/corticosteroids established in humans.

**Method:**

MEDLINE, PubMed, and Embase were systematically searched from inception to December 2023 to identify studies related to IESS aetiology and treatment response. Mechanistic themes were identified and through consensus meetings refined and grouped into five overarching hypotheses.

**Results:**

Five hypotheses were generated from 17 mechanistic themes: (1) gene and epigenetic regulation altering expression of ‘vulnerability’ genes; (2) stress and hypothalamic–pituitary–adrenal axis activation; (3) neuroinflammation and altered immune function; (4) altered neuronal transmission and pathways; and (5) dysfunction of metabolic pathways.

**Interpretation:**

The evidence that ACTH/corticosteroids alter these processes remains limited. It is plausible that these processes interact with one another, rather than existing independently, and affect maturational and regulatory processes in the central nervous system, consistent with proposals that IESS is a neurodevelopmental disorder. Understanding how ACTH/corticosteroids work in IESS may facilitate disease‐modifying treatments and improve neurodevelopmental outcomes.

## Graphical abstract

Why does infantile epileptic spasms syndrome (IESS) occur with a variety of underlying conditions and why does it respond to adrenocorticotrophin hormone (ACTH)/corticosteroids? Our scoping review summarizes five hypotheses from the literature: gene/epigenetic regulation, stress/HPA axis activation, neuroinflammation/immune function, altered neuronal transmission, and metabolic dysfunction. Evidence for ACTH/corticosteroids altering these processes is limited. IESS likely involves interacting processes affecting neurodevelopment. Understanding aetiopathogenic mechanisms further may lead to improved therapies and outcomes.

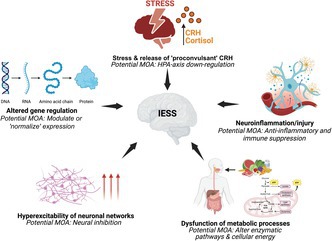

Plain language summary: https://onlinelibrary.wiley.com/doi/10.1111/dmcn.16287

**Abbreviations**
ACTH: adrenocorticotrophin hormone
BBB: blood–brain barrier
CRH: corticotrophin‐releasing hormone
CRHR1: corticotrophin‐releasing hormone receptor type 1
CSF: cerebrospinal fluid
GABA: γ‐aminobutyric acid
GO: gene ontology
HPA: hypothalamic–pituitary–adrenal
IESS: infantile epileptic spasms syndrome
Ig: immunoglobulin
IGF‐1: insulin‐like growth factor 1
IL: interleukin
KYN: kynurenine
KYNA: kynurenic acid
MMP‐9: metallopeptidase‐9
mRNA: messenger RNA
β‐NGF: β‐nerve growth factor
TSC: tuberous sclerosis complex
TNF: tumour‐necrosis factor

**What this paper adds**

- Summarizes five aetiopathogenic mechanisms of infantile epileptic spasms syndrome that adrenocorticotrophin hormone (ACTH)/corticosteroids may modify.
- These include altering gene regulation, stress, neuroinflammation, neuronal excitability, and metabolic pathways.
- Limited reproducible findings in large cohorts constrain the evidence in children.
- Altered gene regulation seems plausible for exploration in future studies of large cohorts.
- Future treatments could target mechanisms underlying this neurodevelopmental disorder rather than one‐off ACTH/corticosteroids.

Infantile spasms is the most common epilepsy syndrome in the first year of life, with an incidence of 3 per 10 000 live births.^1^ Peak onset is between 3 months and 7 months with a slight male predominance.^1^ The syndrome is defined by epileptic spasms, which are clusters of brief tonic contractions of the axial musculature, in addition to developmental arrest or regression of skills with a disorganized, high‐amplitude background on electroencephalogram (EEG) known as hypsarrhythmia.^1^ This triad was previously referred to as ‘West syndrome’; however, some infants may have epileptic spasms with a ‘modified’ hypsarrhythmia or focal EEG abnormalities without developmental changes. These two groups are incorporated by the International League Against Epilepsy as ‘infantile epileptic spasms syndrome’ (IESS).^1^ The aetiology of IESS is highly heterogenous, including more than 200 known causes, classed according to the International League Against Epilepsy as structural, genetic, metabolic, acquired, infectious, and immune aetiologies.^2^ Descriptors such as symptomatic (pre‐, peri‐, post‐natal), cryptogenic, and idiopathic spasms are no longer recommended. It is unclear how multiple aetiologies lead to IESS; however, it is proposed that there is a disruption during a critical developmental period, or multiple insults at various time points causing a ‘desynchronization’ of developmental processes.^3^ It is possible that multiple biological pathways converge or intersect to result in the IESS phenotype.^3^

IESS is often refractory to standard antiseizure medications, and first‐line treatment is hormonal therapy such as intravenous or intramuscular adrenocorticotrophin hormone (ACTH) or high‐dose oral corticosteroids. Vigabatrin is recommended for IESS due to tuberous sclerosis complex (TSC) or may be used in combination with ACTH/steroids for IESS more generally.^4^ Recent meta‐analyses confirm ACTH and corticosteroids have comparable short‐term efficacy, achieving epileptic spasm cessation and resolution of hypsarrthymia in 60% to 70% of children.^5^, ^6^ Corticosteroids offer a cost‐effective and possibly safer alternative to ACTH.^5^, ^7^

Despite worldwide adoption of these aggressive treatment strategies, IESS has a poor long‐term prognosis, 50% to 70% have ongoing epilepsy, 75% are affected by developmental delay/intellectual disability, and 35% to 40% have autism spectrum disorder.^7^ Favourable prognostic factors include an unknown aetiology, typical development before IESS onset, and early initiation and response to treatment.^7^, ^8^, ^9^, ^10^

ACTH and corticosteroids have been used since the 1950s; however, it is not clear how they exert their mechanism of action in the context of IESS. Reviews exploring IESS aetiology and treatment have largely focused on animal models,^11^, ^12^, ^13^ which are inherently limited by their inability to represent a wide range of aetiological mechanisms, generate a typical epileptic spasm with hypsarrthymia on EEG, and use ACTH/corticosteroid formulations generally not prescribed in children. A recent narrative review and cohort study summarized human and animal studies on neurotrophic factors, neurotransmitters, and markers of inflammation to generate therapeutic proposals.^14^ In this review we adopt a systematic approach to explore all published evidence in children with IESS, analysing aetiopathogenesis and the possible mechanism of actions of ACTH/corticosteroids used in clinical practice.

## METHOD

This scoping review was guided by previously described methodological frameworks that emphasize a flexible and iterative search strategy^15^ and written according to the Preferred Reporting Items for Systematic reviews and Meta‐Analyses extension for Scoping Reviews (PRISMA‐ScR).^16^

### Search strategy

To perform this review, MEDLINE, PubMed, and Embase online databases were searched from inception until 20th December 2023. Search terms included a comprehensive list of keywords related to IESS, aetiology, pathophysiology, biomarkers, treatment, and response (further methodology and Medical Subject Headings [MeSH] terms in Appendix S1). To outline genetic causes of IESS, we searched these databases from January 2010 to December 2023 for studies evaluating the yield of genetic testing in IESS cohorts and the associated monogenic and chromosomal disorders (full method in Appendix S2). We only included studies that provided sufficient data to confirm the genetic diagnosis and classify aetiological groups as per International League Against Epilepsy guidelines. Single‐gene studies were excluded. References and citing articles were hand‐searched.

### Eligibility criteria

Eligibility criteria included: (1) a confirmed IESS diagnosis following International League Against Epilepsy definitions; (2) children aged between 0 years and 2 years; (3) aetiology and/or treatment mechanisms explored; (4) ACTH or steroid formulation used in clinical practice for treatment studies; and (5) full‐text journal articles. We excluded studies that: (1) evaluated animal and/or experimental data or; (2) investigated alternative hypotheses, for example insulin tolerance, head circumference, and EEG changes without a clear exploration of underlying biological or treatment effects. Studies were not excluded on the basis of their date of publication and every effort was made to translate publications in languages other than English.

### Study selection and data extraction

Figure S1 outlines the study selection process. The initial search and screening were performed by EAI; duplicates and articles that did not meet eligibility criteria were excluded. EAI, SSM, and RCD reviewed these studies and undertook three consensus meetings to categorize articles into themes describing both aetiological and therapeutic mechanisms (Figure S1).

## RESULTS

**FIGURE 1:**
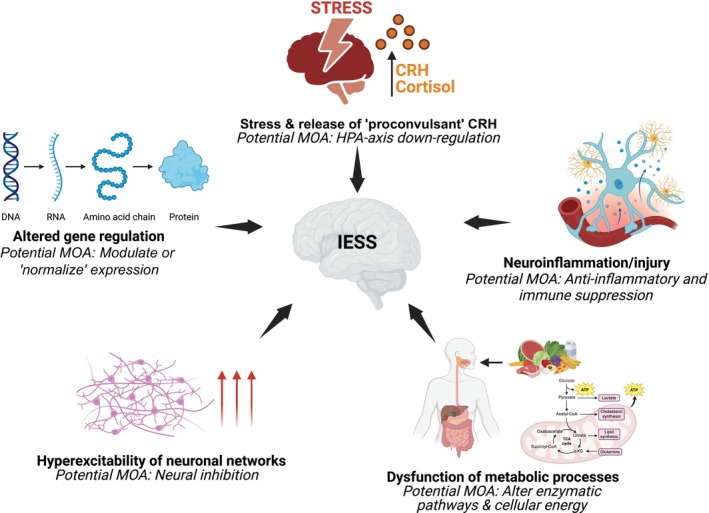
The five aetiopathogenic mechanisms that have been proposed to cause IESS, and the potential mechanism of action of ACTH/corticosteroids. Created with BioRender.com. Abbreviations: CRH, corticotrophin‐releasing hormone; HPA, hypothalamic–pituitary–adrenal; IESS, infantile epileptic spasms syndrome; MOA, mechanism of action.

The search retained 124 studies, which were categorized into 17 themes (Figure S1). These themes could be grouped into five hypotheses (Figure 1): altered gene regulation, stress, neuroinflammation, altered neuronal networks, and altered metabolic pathways. These hypotheses are explored sequentially, focusing on the possible aetiopathogenic mechanisms of IESS and potential antiseizure mechanism of action of ACTH/steroids aligned to each hypothesis. Where possible results were summarized as heat maps to highlight any statistically significant differences in the hormonal, immune, and metabolic profiles of children with IESS compared with controls at baseline and the effect of treatment. Studies without a useful control population or clear pretreatment baseline data were excluded from the heat maps.

### Putative mechanisms underlying IESS and the action of ACTH/steroids

#### Gene variation and gene regulation

This hypothesis was composed of three themes (Figure S1): (1) highly penetrant DNA variations including 24 studies that described pathogenic or likely pathogenic genetic aetiologies for IESS and two studies that analysed biological pathways associated with IESS; (2) DNA polymorphisms explored by nine studies; and (3) one study examining gene regulation and epigenetics. In total, 35 studies explored how altered gene variation/expression may influence IESS onset and whether ACTH/steroids modify gene expression and one study explored epigenetic aspects and gene regulation in the context of IESS.

##### Highly penetrant DNA variation

An aetiological cause for IESS is identified in 56% to 64% of children,^8^, ^17^ leaving a significant proportion ‘unknown’, which are often presumed to be genetic. Twenty‐four studies that describe the genetic aetiology of IESS are outlined in Tables 1–3. These studies are presented in three groups: (1) IESS cohorts including ‘all aetiologies’ to identify the prevalence of aetiological subcategories and yield of genetic testing (Table 1);^17^, ^18^, ^19^, ^20^, ^21^, ^22^, ^23^, ^24^ (2) IESS with ‘presumed/confirmed’ genetic aetiology and yield of testing (Table 2);^25^, ^26^, ^27^, ^28^, ^29^, ^30^, ^31^, ^32^, ^33^, ^34^, ^35^, ^36^, ^37^, ^38^, ^39^ and (3) IESS with confirmed genetic diagnoses (Table 3).^40^

**TABLE 1:** Studies of IESS which included all aetiologies and subgroups are presented (eight studies with total 2116 cases), including inclusion/exclusion criteria, the breakdown of aetiological subgroups, genetic testing used and yield, and the monogenic causes found.

Study	IESS cohort (*n*)	Selection criteria	Aetiological subgroups (*n*, %)	Method and yield of genetic testing (*n*, %)	Genes identified by aetiological subgroup (*n*, % of total cohort)
Peng et al.^19^	541	Included: 0–2 years Excluded: nil	Unknown (253, 47%) Structural–acquired (137, 25%) Genetic (70, 13%) Genetic–structural (39, 7%) Structural–congenital (27, 5%) Metabolic (13, 2%) Infection (2, 0.4%)	Total: 105 out of 541 (19%) CMA: 12 out of 207 (6%) Karyotype: 2 out of 183 (1%) Epileptic encephalopathy panel: 27 out of 105 (26%) WES: 63 out of 234 (27%) Mitochondrial genome analysis: 1 out of 24 (3%)	**Genetic (70, 13%):** Single‐gene disorders (59, 11%): 25 genes *Pathogenic/likely pathogenic: STXBP1* (12), *CDKL5* (12), *KCNQ2* (5), *IRF2BPL* (4), *CLCN4* (2), *GNAO1* (2), *SCN8A* (2), *KCNB1* (2), *SCN2A* (2), *CYFIP2* + *KMT2D* (1), *DNM1* (1), *ARX* (1), *GRIN2B* (1), *AARS* (1), *NTRK2* (1), *STPAN1* (1), *GNB1* (1), *KMT2D* (1), *SMARCA2* (1) *CLCN4 (1)*, *SCN10A (1)*, *CACNA1A (1)*, *GABRE (1)*, *UFC1 (1)* Chromosomal disorders/CNV (11, 2%): 8 genes *Pathogenic/likely pathogenic:* 1p36.33del (2) 1p36.33‐32del (1), Xp22.11–21.3dup (1), Xp22.13del (1), 20q13.33del (1), 15q11.2dup (1), 9q33.3–34.11 del (1), 9p24.3–22.del (1) 5p12‐11dup (1), 3p25.3del (1) Genes involved: *CDKL5*, *EEF1A2*, *KCNQ2*, *STXBP1*, *HCN1*, *SETD5*, *GNB1*, *ARX* **Genetic–structural (39, 7%): 6 genes** Single‐gene disorders *Pathogenic/likely pathogenic: TSC2* (10), *TSC1* (4), TSC, no gene (17), *NF1* (2), NF, no gene (2), *NEDD4L* (1), *DCX* (1), *NPRL3* (1), Chromosomal: 17p13.3del (1) **Metabolic (13, 2%): 10 genes** *Pathogenic/likely pathogenic: WDR45* (3), *SLC35A2* (1), *ALG1* (1), *ALG13* (1), *ATP7A* (1), *MMACHC* (1), *ACADS* (1), *SLC35A2 (1)*, *HEXA* (1), *ALDH7A1* (1) Mitochondrial: *MT*‐*ND1* (1)
Liu et al.^18^	728	Included: 0–2 years Excluded: nil^a^	Unknown (442, 61%)^b^ Genetic–structural (129, 18%) Structural (79, 11%) Genetic (76, 10%) Metabolic (2, <1%)	Total: 185 out of 728 (25%) CMA/epileptic encephalopathy /WES: 75 out of 442 (17%) Karyotype: 1 out of 1 (100%) Targeted gene PCR: 108 out of 129 (84%) Epileptic encephalopathy panel: 0 out of 13 (0%) Metabolic panel: 2 out of 2 (100%)	**Genetic (76, 10%):** Single‐gene disorders (62, 9%): 27 genes *Pathogenic/likely pathogenic: STXBP1* (21), *SCN2A* (6), *CDKL5* (6), *ARX* (2), *KCNQ2* (2), *KCNB1* (2), *RARS* (2), *RYR3* (2), *DIAPH3* + *STXBP1* (1), *ALG13* (1), *CACNA1A* (1), *GRIN2B* (1), *IQSEC2* (1), *KCNMA1* (1), *MEF2C* (1), *NID2* (1), *SCN8A* (1), *TCF4* (1), *SHANK3* (1), *ALDH7A1* (1), *ASAH1* (1), *CUBN* (1), *DOCK7* (1), *NRXN1* (1), *TBC1D24* (1), *TNK2* (1), *VRK* (1) Chromosomal (14, 2%): 7 genes 1p36 del (2), 17pdel (2), 7q11del (2), 15q Dup (1), trisomy 21 (1), other (6) Genes involved: *UBE3A*, *GABRB3*, *CHRNA7*, *NDE1*, *MYH11*, *GABRD*, *MAGI2* **Genetic–structural (129, 18%): 3 genes:** *TSC2* (91), *TSC1* (15), TSC‐no gene (21), *NF1* (2)
Jiang et al.^20^	441	Included: 2 months‐2 years Excluded: no MRI, ACTH or vigabatrin treatment	Unknown (223, 51%) Structural–acquired (104, 24%) Genetic (68, 15%) Congenital–structural (31, 7%) Infection (10, 2%) Metabolic (5, 1%)	Total: 68 out of 441 (15%) CMA: N/A Karyotype: N/ A Epileptic encephalopathy panel: N/ A WES: N/ A	**Genetic (68, 15%):** Single‐gene disorders (68, 15%): 24 genes *Pathogenic: TSC1/2* (30), *CDKL5* (4), *KCNQ2* (4), *SCN2A* (4), *DEPDC5* (3) *COL4A1* (2), *FOXG1* (2), Other (19)‐ N/A Chromosomal disorders (0%)
Yan et al.^21^	41	Included: >1 year //‐ 3.5 years Excluded: <1 year	Structural (21, 51%) Unknown (11, 27%) Genetic (9, 22%)	Total: 9 out of 41 (22%) WES: 9 out of 21 (31%)	**Genetic (9, 22%):** Single‐gene disorders (9, 22%): 7 genes *Pathogenic/likely pathogenic: POLR2A* (1), *SCN2A* (1), *WDR45* (1), *SCN1A* (1), *GRIA2* (1), *GTPBP3* (1), *CDKL5* (1), *ADA2* (1) Mitochondrial: chrM‐144485 T > C, ChrM‐3736 M > A Chromosomal disorders (0%)
Demarest et al.^23^	21	Included: 0–1 year, had ACTH treatment, referred for WES Excluded: nil	Genetic and genetic–structural (12, 57%) Unknown (7, 33%) Structural–acquired (2, 10%)	Total: 5 out of 21 (24%) CMA: 0 out of 13 Epileptic encephalopathy panel: 0 out of 11 WES (trio): 5 out of 21 (24%)	**Genetic and genetic–structural (12, 57%):** Single‐gene disorders (10, 48%): 10 genes *Pathogenic*: *NR2F1* (1), *GNB1* (1), *NEUROD2* (1), *NDUFAF5* (1) *GABRA2* (1) *VOUS*: *PEMT* (1), *ASXL2* (1), *RALGAPB* (1), STRADA (1), *DYNC1I1* (1) Chromosomal disorders (2, 10%) Trisomy 21 (2)
Symonds et al.^22^	52	Included: 0–3 years^c^ Excluded: nil	Unknown (16, 31%) Structural (14, 27%) Genetic–structural (12, 24%) Genetic (9, 17%) Genetic–metabolic (1, 1%)	Total: 22 out of 52 (42%) CMA: N/A Epileptic encephalopathy panel: N/ A WGS (trio): N/ A	**Genetic, genetic–structural and metabolic (22, 42%):** Single‐gene disorders (13, 25%): 9 genes *Pathogenic/likely pathogenic: TSC2* (3), *TSC1* (2), *CDKL5* (2) *IDIC15* (1), *ASAH1* (1), *CACNA1G* (1), *DEPDC5* (1), *PAFAH1B1* (1), *POLR1A* (1) Chromosomal disorders/CNV (9, 17%) Trisomy 21 (6), trisomy 13 (1) 16p13.11 del (1), 17p13.3 del (Miller–Dieker) (1)
Helbig et al.^24^	41	Included: 0–18 years^c^ referred for WES Excluded: nil	Genetic (16, 39%) Other diagnoses N/A	Total: 16 out of 41 (39%) WES (singleton or trio): 16 out of 41 (39%)	**Genetic (16, 39%):** Single‐gene disorders (16, 39%): 18 genes *Pathogenic/likely pathogenic: FOXG1* (2), *CACNA1C* (1), *CCND2* (1), *COL4A1* (1), *DYNC1H1* (1), *GNAO1* + *ATP2B3* (1), *KMT2A* (1), *RARS2* (1), *ANO3* + *NALCN* (1), *DNM1* (1) *VOUS (possibly pathogenic): CACNA1E* (1), *AIMP2* + *LRFN2* (1), *COQ4* (1), *HNRNPR* (1), *HTR2C* (1)
Wirrell et al.^17^	251	Included: 0–2 years Excluded: nil	Unknown (90, 36%) Structural–acquired (55, 22%) Genetic (36, 14%) Structural–congenital (27, 11%) Genetic–structural (25, 10%) Metabolic (12, 5%) Infection (5, 2%)	Total: 57 out of 251 (23%) CMA: 12 out of 87 (14%) Karyotype: 17 out of 32 (53%) Chromosomal SNP: 2 out of 4 (50%) Targeted gene SNP: 11 out of 24 (46%) Epileptic encephalopathy panel: 11 out of 34 (32%) WES//WGS: 0 out of 4 (0%) Mitochondrial SNP: 1 out of 4 (25%) Mitochondrial gene panel: 3 out of 9 (33%)	**Genetic (36, 14%):** Single‐gene disorders (11, 4%): 7 genes *Pathogenic/likely pathogenic: CDKL5* (3), *KCNQ3* (2) *LYK5* (2), *STXBP1* (1), *SCN1A* (1), *KRAS* (1), *KANSL* (1) Chromosomal disorders/CNV (25, 10%) *Pathogenic/likely pathogenic:* risomy 21 (15), 15q11 mut (3) chr2 mut (2), Williams syndrome (1), trisomy 13 (1) 15q21 mut (1), chr6 mut (1), other (1) **Genetic–structural (25, 10%): 7 genes** *Pathogenic/likely pathogenic: TS1/2 (*12), *NF1* (2), *DCX* (1), *LIS1* (1) Miller–Dieker (1), *AGS* (1), *SETBP1* (1) 15q11.2 dup (1), cortical malformations with genetic change (5) **Metabolic (12, 5%): 3 genes** *Pathogenic/likely pathogenic: POMPT1* (1), *POLG1* (1), Leigh disease, *ATP6* (1)
	Total cohort 2116			Positive genetic result Total: 467 out of 2116 (22%) Mean: 26% Median: 23% Range: 15–42%	

^a^ No genetic testing for acquired cases.

^b^ Seventy‐two had no genetic testing.

^c^ Part of DEE cohort.

Abbreviations: ACTH, adrenocorticotrophin hormone; CMA, chromosomal microarray; CNV, copy number variant; DEE, developmental and epileptic encephalopathy; FCD, focal cortical dysplasia; IESS, infantile epileptic spasms syndrome; MRI, magnetic resonance imaging; N/A, not available; NF, neurofibromatosis; PCR, polymerase chain reaction; SNP, single‐nucleotide polymorphism; TSC, tuberous sclerosis complex; VOUS, variant of uncertain significance; WES, whole‐exome sequencing; WGS, whole‐genome sequencing (singleton, duo, or trio, stated if known).

**TABLE 2:** Studies of IESS which included presumed/confirmed genetic causes of IESS (15 studies with total 936 cases), including inclusion/exclusion criteria, genetic testing used and yield, and the monogenic causes found.

Study	IESS cohort (*n*)	Selection criteria	Method and yield of genetic testing	Genes identified by aetiological subgroup (*n*, % of total cohort)
D'Gama et al.^25^	32	Included: 0–1 year^b^ Excluded: known genetic, acquired causes	Total: 6 out of 32 (19%) WGS (singleton, duo, or trio): 6 out of 32 (19%)	**Genetic (6, 19%):** Single‐gene disorders (5, 16%): 5 genes *Pathogenic/likely pathogenic: STXBP1* (1), *PTEN* (1), *TED5* (1), *KCNJ6* (1), *DYNC1H1* (1) Chromosomal disorders/CNV (1, 3%) 9pterq22.23 15q22.2qter 9
Koh et al.^26^	46	Included: 0–18 years^b^, IESS onset < 1 year Excluded: known genetic, acquired causes	Total: 9 out of 46 (20%) WGS (singleton, duo, or trio): 9 out of 46 (20%)	**Genetic and genetic–structural (9, 20%):** Single‐gene disorders (8, 17%): 8 genes *Pathogenic/likely pathogenic: STXBP1* (1), *CYFIP2* (1), *DYNC1H1* (1), *GRIN2B* (1), *NPRL2* (1), *OTUD6B* (1), *PGAP2* (1) *VOUS: MTR* (1) Chromosomal disorders/CNV (1, 2%): Chr16:138446–140150
Scheffer et al.^27^	38	Included: 0–18 years^b^, IESS onset presumed < 2 years, negative CMA Excluded: known genetic, structural, causes	Total: 4 out of 38 (11%) WES (singleton, duo, or trio): 4 out of 38 (11%)	**Genetic (4, 11%):** Single‐gene disorders (3, 8%): 3 genes *Pathogenic/likely pathogenic: STXBP1* (1) *ALG13* (1), *HECW2* (1) Chromosomal disorders/CNV (1, 3%): 1 gene 9q33.3q34.11 del (*STXBP1*)
Lee et al.^28^	16	Included: 0–1 year Excluded: known structural, acquired, metabolic causes	Total: 4 out of 16 (25%) WGS (singleton or trio): 4 out of 16 (25%)	**Genetic (4, 25%):** Single‐gene disorders (4, 25%): 4 genes *Pathogenic/likely pathogenic: HDAC4 (1)*, *GRM7 (1)*, *CACNA1E (1)*, *KMT2E (1) new line before VOUS*: SOX5 (1), SHROOM4 (1)
Choi et al.^29^	58	Included: 0–1 year Excluded: known genetic cause (from CMA/karyotype), structural, metabolic causes	Total: 17 out of 58 (29%) Epileptic encephalopathy panel: 17 out of 58 (29%)	**Genetic (17, 29%):** Single‐gene disorders (14, 24%): 7 genes *Pathogenic: CDKL5* (4), *STXBP1* (3), *KCNB1* (2), *SCN2A* (2), *EEF1A2* (1), *KANSL1* (1), *MECP2* (1) CNV (3, 5%): not specified
Krey et al.^30^	45	Included: 0–40 years, IESS onset < 1 year Excluded: TSC, trisomy 21, structural–acquired causes	Total: 13 out of 45 (29%) Epileptic encephalopathy panel: 13 out of 45 (29%)	**Genetic (13, 29%):** Single‐gene disorders (11, 24%): 7 genes *Pathogenic/likely pathogenic: CDKL5* (5), *ARX* (1), *SCN1A* (1), *KCNB1* (1), *DEPDC5* (1), *AARS* (1), *WDR45* (1) Chromosomal disorders/CNV (2, 4%): 20 genes 2q24.1q24.3 dup (1), 15q11.1q13.1 trip (1) Genes involved: *GABRB3*, *TUBGCP5*, *NIPA1*, *MKRN3*, *MAGEL2*, *NDN*, *SNRPN*, *UBE3A*, *ATP10A*; *SCN2A*, *SCN3A*, *CD302*, *FAP*, *IFIHI*, *GCA*, *KCNH7*, *FIGN*, *GRB14*, *COBLL1*, *TANK*
Muir et al.^31^	92	Included: 0–2 years Excluded: known structural, metabolic causes	Total: 7 out of 92 (8%) Epileptic encephalopathy panel: 7 out of 92 (8%)	**Genetic (7, 8%):** Single‐gene disorders (7, 8%): 6 genes *Pathogenic: KCNB1* (2), *GNAO1* (1), *KIF1A* (1), *SLC35A2* (1), *STXBP1* (1), and *TBL1XR1* (1) *VOUS*: *FASN* (1), *HDAC4* (1), *PNMAL1* (1), *PPP3CA* (1)
EPGP/ Yuskaitis et al.^32^	133	Included: 0–1 year, 6 months follow‐up Excluded: known genetic, structural, metabolic causes, severe developmental delay before IESS	Total: 15 out of 100 (15%) WES (trio): 15 out of 100 (15%)	**Genetic (15, 15%):** Single‐gene disorders (15, 15%): 12 genes *Pathogenic: STXBP1* (3), *KCNQ2* (2), *ALG13* (1), *DNM1* (1), *GABRA1* (1), *GNAO1* (1), *GRIN1* (1), *KCNT1* (1), *PTEN* (1), *SCN2A* (1), *SCN8A* (1), *TUBB2A* (1)
Ko et al.^33^	128	Included: 0–3 years^a^ normal CMA, cause confirmed by epileptic encephalopathy NGS panel Excluded: structural, metabolic causes	Total: 34 out of 128 (27%) Epileptic encephalopathy panel: 34 out of 128 (27%)	**Genetic (34, 27%):** Single‐gene disorders (34, 27%): 14 genes *Pathogenic/likely pathogenic: CDKL5 (7)*, *STXBP1 (6)*, *SCN8A (5)*, *KCNQ2 (3)*, *KCNB1 (2)*, *DNM1 (2)*, *SCN2A (2)*, *ARX (1)*, *WWOX (1)*, *BRAT (1)*, *EEF1A2 (1)*, *HCN1 (1)*, *MECP2 (1)*, *PRODH (1)*
Dimassi et al.^34^	10	Included: 0–1 year, negative CMA, negative *CDKL5*, *STXBP1* and *ARX* Excluded: known genetic, metabolic structural, acquired causes, family history of seizure, consanguinity	Total: 4 out of 10 (40%) WES (trio): 4 out of 10 (40%)	**Genetic (4, 40%):** Single‐gene disorders (4, 40%): 4 genes *Possibly pathogenic: CDKL5* (1), *STXBP1* (1), *ALG13* (1), *NR2F1* (1) Chromosomal disorders/CNV (0)
Boutry‐Kryza et al.^35^	73	Included: 0–11 years, IESS onset <2 years, negative CMA, karyotype, *ARX* testing Excluded: known genetic, structural, metabolic, acquired causes	Total: 11 out of 73 (15%) CMA and SNP analysis for 5 genes (*CDKL5*, *STXBP1*, *KCNQ2*, *GRIN2A*, *MAGI2):* 11 out of 73 (15%)	**Genetic (11, 15%):** Single‐gene disorders (6, 8%): 2 genes *Pathogenic/likely pathogenic: CDKL5* (3), *STXBP1* (3) Chromosomal disorders/CNV (5, 7%): 30 genes *Pathogenic*: Xq27.1 dup (1), 2q22.3 q24.2 del (1), 5q14.3 del (1), 9q3.3 del (1), 2q24.2 dup (1) Genes involved: *MIR3660*, *CETN3*, *LOC731157*, *MBLAC2*, *POLR3G*, *LSMD3*, *GPR98*, *LUCAT1*, *ARRDC3*, *EHMT1*, *CACNA1B*, *SCN2A*, *CSRNP3*, *GLANT3*, *TTC21B*, *NEDD4*, *CALN1*, *OTOA*, *RRN3P1*, *UQCRC2*, *PDZD9*, C16orf52, *VWA3A*, *EEF2K*, *POLR3E*, *CDR2*, *RRN3P3*, *SMG1P1*, *LOC653786*, *NPIPB5* *Potential risk factor:* (3)‐see original
Hino‐Fukuyo et al.^36^	18	Included: 0–2 years Excluded: known genetic, structural, metabolic, acquired causes; neonatal onset IESS	Total: 9 out of 18 (50%) CMA: 4 out of 18 (22%) Epileptic encephalopathy panel: 0 out of 14 (0%) WES (trio): 5 out of 14 (36%)	**Genetic (9, 50%):** Single‐gene disorders (5, 27%): 5 genes *Pathogenic/likely pathogenic: SLC35A2* (1), *NR2F1* (1), *CACNA2D1* (1), *ALG13* (1), *BRWD3* (1) Chromosomal disorders/ CNV (4, 22%): > 100 genes *Likely pathogenic*: Xq28 dup (1), 19p13.2 del (1), *Possibly pathogenic*: 16p13.1 del (1), 19p13.2 del (1) *Benign* (5)
Michaud et al.^37^	44	Included: 0–2 years, non‐specific MRI brain findings Excluded: structural, metabolic, causes	Total: 11 out of 44 (25%) CMA: 4 out of 44 (9%) Epileptic encephalopathy panel: 0 out of 8 (0%) Targeted sequencing (*ARX/* *CDKL/ STXBP1*): 2 out of 38 (5%) WES (trio): 5 out of 18 (28%)	**Genetic (11, 25%):** Single‐gene disorders (7, 16%): 7 genes *Pathogenic: STXBP1* (2), *ARX* (1), *PNPO* (1), *ADSL* (1), *CASK* (1) *ALG13* (1) Eleven new candidate genes (8, 18%) predicted pathogenic *SQSTM1*, *MYO9B*, *NR2F1*, *NPC1L1*, *TNFA1P6*, *EIF2C4*, *TENM2*, *HEG1*, *LAMA2*, *SEMA5B*, *HSPG2* Chromosomal disorders/CNV (4, 9%): 5 genes *Likely pathogenic:* 2q21.3‐q22.1 del (1), 15q11.1q13.1 tetrasomy (1), 16p11.2 dup (1) Genes involved: *CXCR4*, *NXPH2*, *GABRA5*, *GABRA3*, *UBE3A*
EuroEPIGENOMICS, Epi4K, EPGP et al.^38^	159	Included: 0–2 years^a^ Excluded: structural, metabolic, acquired causes, family history of epilepsy	Total: 42 out of 159 (26%) WES (trio): 42 out of 159 (26%)	**Genetic:** **Single‐gene disorders (42, 26%): 46 genes** *De novo probably damaging: STXBP1* (3), *CDKL5* (1), *CNTN5* (1), *GABRA1* (1), *GABRB1* (1), *GABRB3* (1), *GRIN1* (1), *KCNB1* (1), *SCN8A* (1), *KCNQ2* (1), *SCN2A* (1), *TIFA* (1), *FAM86C1* (1), *SLC16A3* (1), *PNMAL1* (1), *PIK3AP1* (1), *NLRP8* (1), *TRRAP* (1), *SMURF1* (1), *C1QTNF6* (1), *ATP2B4* (1), *SLAMF1* (1), *GNAO1* (1), *OR10S1* (1), *NGLN2* (1), *PLA1A* (1), *FAM50A* (1), *ASXL1* (1) *DHTKD1* (1), *TAF1* (1), *ETS1* (1), *TRIM29* (1), *AKR1C4* (1), *CSNK1E* (1), *KIAA2018* (1), *ITGAM* (1), *PRDM12* (1), *THOC2* (1), *SMG9* (1), *DNM1* (1), *NR1H2* (1), *HIST2H2BE* (1), *SLC1A2* (1), *RRP1B* (1), *ANKRD12* (1) *De novo possibly damaging: AGL13* (1), NCOR2 (1), *KCNT1* (1), *NFASC* (1), *SGK223* (1), *C1QTNF6* (1), *NLRP5* (1), *TNNI3K* (1) *PALLD* (1), *MSANTD1* (1), *RFX3* (1), *GAS2* (1), *DIAPH3* (1), *PACS2* (1), *NEDD4L* (1), *PDIK1L* (1)
Mefford et al.^39^	44	Included: 0–2 years^a^ Excluded: known causes (various physician‐directed investigations)	Total: 3 out of 44 (7%) CMA: 3 out of 44 (7%)	**Genetic (3, 7%):** Chromosomal disorders/CNV (3, 7%): 4 genes *Pathogenic/likely pathogenic:* Xp22 del, 16p11.2dup Genes involved: *LRRK2*, *SCLT1*, *EPHA6*, *GABRR3* VOUS: 12q12del and 4q28del, 3q11 dup
	Total cohort 936		Positive genetic result Total: 189 out of 936 (20%) Mean: 23% Median: 23% Range: 7–50%	

^a^ Part of DEE cohort.

Abbreviations: ACTH, adrenocorticotrophin hormone; ASD, autism spectrum disorder; CMA, chromosomal microarray; CNV, copy number variant; DEE, developmental and epileptic encephalopathy; FCD, focal cortical dysplasia; IESS, infantile epileptic spasms syndrome; MRI, magnetic resonance imaging; N/A, not available; NF, neurofibromatosis; NGS, next‐generation sequencing; SNP, single‐nucleotide polymorphism; TSC, tuberous sclerosis complex; VOUS, variant of uncertain significance; WES, whole‐exome sequencing; WGS, whole‐genome sequencing (singleton, duo, or trio, stated if known).

**TABLE 3:** Studies of IESS reporting confirmed genetic causes (one study with 124 cases), including inclusion/exclusion criteria and monogenic causes found.

Study	IESS cohort (*n*)	Selection criteria	Genes identified by aetiological subgroup (*n*, % of total cohort)
Nagarajan et al.^40^	124	Included: 2 months to 2 years, genetic cause confirmed by CMA, karyotype, triplet repeat PCR or methylation MLPA, Sanger sequencing, or NGS Excluded: structural–genetic, metabolic causes	**Genetic (124, 100%):** Single‐gene disorders (105, 85%): 51 genes *Pathogenic/likely pathogenic: ALDH7A1* (10), *SCN2A* (7), *CDKL5* (6), *ALG13* (5), *KCNQ2* (4), *STXBP1* (4), *WWOX* (4), *SCN1A* (4), *NTRK2* (4), *KCNT1* (3), *SYNGPA1* (3), *SCN3A* (3), *SLC2A1* (3), *MECP2* (2), *CPLX1* (2), *UGP2* (2), *PP3CA* (2), *PLPBP* (2), *GRM7* (1), *TBCD* (1), *CHD2* (1), *CDK19* (1), *FOXG1* (1), *NRROS* (1), *PURA* (1), *KANSL1* (1), *GABBR2* (1), *GRIN1* (1), *CSNK2A1* (1), *PNPO* (1), *CACNA1A* (1), *NPRL3* (1), *IQSEC2*, (1), *CYFIP2* (1), *MBOAT7* (1), *MBD5* (1), *PPP2R1A* (1), *DNM1* (1), *NONO* (1), *EHMT1* (1), *GNAO1* (1), *PRRT2* (1), *AMT* (1), KMT2C (1), *ADSL* (1), *SATB1* (1), *PACS2* (1), *HUWE1* (1), *ASNS* (1), *MIPEP* (1), *PLEKHG2* (1), *SCN8A* (1) Mitochondrial: *likely pathogenic MT‐ND5* (1) Chromosomal disorders/CNV (19, 15%) *Pathogenic*: trisomy 21 (14), Xq28 dup (2), cri‐du‐chat (1), 15q dup (1), 1p36 del 18 g dup (1)

Abbreviations: CMA, chromosomal microarray; CNV, copy number variant; DEE, developmental and epileptic encephalopathy; FCD, focal cortical dysplasia; IESS, infantile epileptic spasms syndrome; MLPA, multiplex ligation‐dependent probe amplification; NGS, next‐generation sequencing; PCR, polymerase chain reaction.

**FIGURE 2:**
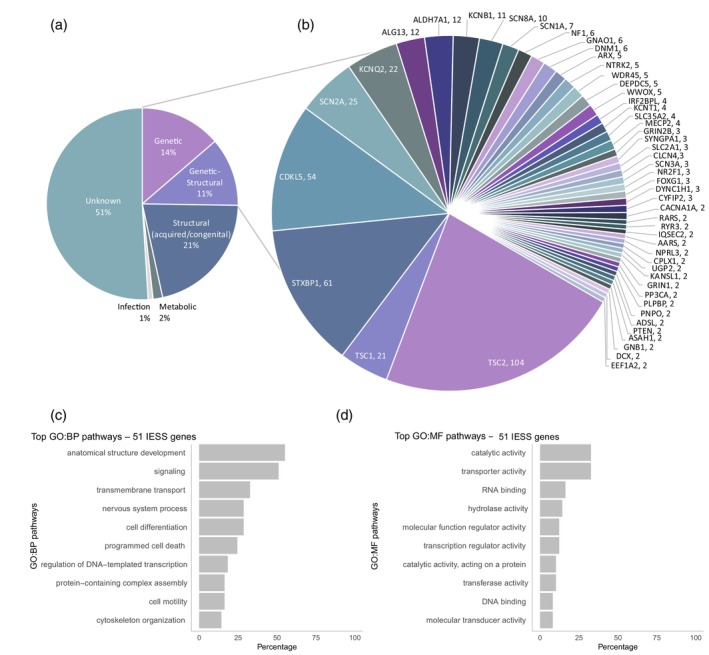
The aetiologies of IESS in studies of IESS (Tables 1, 2, 3). (a) The combined prevalence of aetiological subgroups in IESS combined from Table 1: eight studies with 2116 children (including all known aetiologies). Aetiology was classified as per International League Against Epilepsy guidelines. (b) Prevalence of 51 pathogenic/likely pathogenic genes associated with IESS identified in two or more individuals from 25 studies with 3176 children (combined from Tables 1, 2, 3). Note: a small proportion of cases with TSC and NF were excluded, as no gene was identified or *TSC1* was not differentiated from *TSC2*. (c, d) These 51 genes were categorized using GO slim BP and MF terms. The GO Term Mapper from the Lewis‐Sigler Institute for integrative genomics was used and graphs were generated using the ggplot2 package on the R platform (R Foundation for Statistical Computing, Vienna, Austria). The top 10 GO BP pathways enriched by the genes included anatomical structure development (55% of genes), signalling (51% of genes), and transmembrane transport (33% of genes). The top 10 GO MF pathways enriched by the genes included catalytic activity (33% of genes), transporter activity (33% of genes), and RNA binding (16% of genes). Abbreviations: BP, biological processes; GO, gene ontology; IESS, infantile epileptic spasms syndrome; MF, molecular function.

From IESS studies examining ‘all aetiologies’, 51% of 2116 children had ‘unknown aetiology’, as presented in Figure 2a. This is probably a reflection of the variability of investigations performed (details in Table 1). The second largest aetiological group was structural (acquired/congenital) disorders (21%) followed by genetic (14%), genetic–structural (11%), metabolic (2%), and infectious (1%) aetiologies. The diagnostic yield from all genetic tests combined was 22% (467 out of 2116) in IESS including ‘all aetiologies’ (Table 1), and 20% (189 out of 936) in IESS ‘presumed/confirmed genetic aetiology’ (Table 2). The yield of individual tests was similar in ‘all aetiology’ and ‘presumed/confirmed genetic aetiology’ cohorts: chromosomal microarray (8% vs 9%), epileptic encephalopathy panel (23% vs 25%), and whole‐exome/genome sequencing (29% vs 21%). Karyotyping was diagnostic in 6% of the IESS ‘all aetiology’ cohort, with trisomy 21 the most prevalent chromosomal disorder.

Figure 2b demonstrates the significant genetic heterogeneity of monogenic disorders associated with IESS. On the basis of our review, we identified 51 genes that were reported as pathogenic/likely pathogenic in two or more individuals from the IESS cohorts in Tables 1–3. The 10 most prevalent monogenic disorders were *TSC2* (22% of all monogenic cases), *TSC1* (5%), *STXBP1* (13%), *CDKL5* (12%), *SCN2A* (5%), *KCNQ2* (5%), *ALG13* (3%), *ALDH‐7A1* (3%), *KCNB1* (2%), and *SCN1A* (2%). We further performed a gene ontology (GO) pathway analysis^41^ using slim terms to identify the key biological processes (Figure 2c) and molecular functions (Figure 2d) shared by these 51 genes (method summarized in Figure 2). The top GO biological processes pathways enriched by these genes included anatomical structural development, signalling, and transmembrane transport whereas the top GO molecular functions pathways involved catalytic and transporter activity and RNA binding.

A previous study used GO pathway analysis to compare pathogenic variants in IESS to other infantile epilepsies:^42^ the GO pathways enriched by genes in IESS included broad immunological, developmental, and regulatory pathways of the central nervous system (CNS) including cell cycle regulation and tumorigenic processes (microRNA regulation).^42^ Another study performed GO pathway analysis and protein–protein interaction networks to investigate pathways enriched by genes in novel copy number variants in IESS.^43^ Pathways over‐represented involved ventral forebrain development and synaptic function (transport and signalling pathways).^43^ Haploinsufficiency was examined; however, the pathogenicity of certain novel microduplications was not able to be assigned.^43^

##### DNA polymorphisms

DNA polymorphisms, or single‐nucleotide polymorphisms, are the most common type of genetic variation, which may confer susceptibility or resistance to disease. Single‐nucleotide polymorphisms are defined by their presence in more than 1% of the population.^44^ Investigators have pursued hypotheses that polymorphisms in genes encoding the glucocorticoid receptor (*NRC3C1*), melanocortin receptor (*MC2R*, *MC3R*, *MC4R*), and corticotrophin‐releasing hormone receptor (*CRH1*) could affect the stress response by increasing the risk of IESS or modifying response to ACTH.^45^, ^46^, ^47^, ^48^, ^49^ Polymorphisms of genes involved in neurotransmission were also studied including *SCN1A* (encoding sodium channels), *GRIN1* (*N*‐methyl‐d‐aspartate, NMDA receptor), *5‐HTT* (serotonin transporter), and *ABCB1*, a gene encoding P‐glycoprotein, an efflux pump of the blood–brain barrier (BBB).^50^, ^51^, ^52^, ^53^ Although single‐nucleotide polymorphisms in *MC2R*, *MC4R*, *NRC3C1*, *SCN1A*, *GRIN1*, and *5‐HTT* were associated with ACTH response,^50^, ^51^, ^52^ the significance of these findings is unclear and limited by their targeted hypothesis‐driven approach (as opposed to genome‐wide association studies) and lack of replication by other studies.

##### Gene regulation and epigenetics

Gene regulation can be analysed through RNA sequencing, proteomics, and epigenetic studies. No studies were identified that have used untargeted RNA or proteomic sequencing in blood or brain tissue to identify aetiological or therapeutic mechanisms in IESS.

Epigenetic regulation involves chemical modifications to the genome that switches genes ‘on’ or ‘off’ without altering the DNA sequence. This includes DNA methylation, histone modification, or altering non‐coding RNAs. Epigenetic modifications can be induced by stress and environmental and lifestyle factors. One study demonstrated lower global DNA methylation in lymphocytes of children with IESS of unknown aetiology, compared with typically developing controls.^54^ Pretreatment methylation levels did not differ between ACTH responders and non‐responders; however, samples were not re‐collected to determine whether ACTH altered methylation.

#### Hypothalamic–pituitary–adrenal axis

This hypothesis consisted of three themes and 24 studies that described hypothalamic–pituitary–adrenal (HPA)‐axis hormones to determine whether the stress system is activated at IESS onset and whether ACTH/steroids modify the stress response. Five studies examined hypothalamic hormones, 13 studied pituitary and thyroid hormones, and 15 examined adrenal hormones, with several studies overlapping (Figure S1).

##### Hypothalamic hormones (corticotrophin‐releasing hormone, somatostatin)

Three studies examined the hypothesis that there is excessive release of ‘stress’ hormones in IESS, particularly the ‘pro‐convulsant’ corticotrophin‐releasing hormone (CRH), known to trigger seizures in animal studies.^55^

One study examined messenger RNA (mRNA) and protein expression of CRH and CRH receptor type 1 (CRHR1) in brain tissue of children with IESS.^56^ Tissue was resected during epilepsy surgery and compared with autopsy controls. In IESS, CRH and CRHR1 were significantly upregulated.^56^ No studies measured CRH levels or CRH mRNA expression in blood. Two studies quantified CRH levels in cerebrospinal fluid (CSF) compared with controls undergoing CSF testing for fever, seizures, or neurological conditions (Figure S2). CSF CRH level was no different in IESS compared with controls.^57^, ^58^ One study found CSF CRH inversely correlated with low CSF cortisol, indicating possible suppression of downstream ‘stress’ hormones.^57^ No studies have analysed the effect of treatment on CRH.

Two studies examined CSF somatostatin, which is an excitatory neurotransmitter. CSF somatostatin was higher in IESS than controls in one study^59^ and similar to controls in another.^60^ ACTH reduced CSF somatostatin levels, but not significantly.^59^, ^60^ Blood somatostatin levels have not been measured in IESS.

##### Pituitary and thyroid hormones

Several studies have quantified ACTH in blood and CSF, hypothesizing that IESS results from an ACTH deficiency which treatment corrects.^61^, ^62^, ^63^, ^64^ Authors have proposed a primary and/or secondary ACTH deficiency due to stress.^64^

At IESS onset, blood ACTH levels were no different to controls,^62^ or within a normal range (Figure S3).^65^, ^66^ In contrast, five studies reported CSF ACTH levels were significantly lower in IESS than controls^57^, ^58^, ^61^, ^62^, ^67^ (Figure S2) and lowest in IESS secondary to known aetiologies (e.g. brain injury, trisomy 21).^63^, ^68^, ^69^ ACTH treatment did not ‘correct’ the CSF ACTH deficiency^61–63,69^ or lead to a significant increase in blood ACTH (Figures S2 and S3).^65^, ^66^

β‐Endorphin was investigated as a surrogate marker of ACTH, as they share the same precursor hormone, pro‐opiomelanocortin. Most studies found no differences in blood^62^ or CSF β‐endorphin levels^61^, ^62^ between IESS and controls. Only one study found CSF β‐endorphin was significantly lower in IESS.^58^ In a separate study, ACTH treatment led to a non‐significant reduction of CSF β‐endorphin (Figure S2).^69^

At IESS onset, other pituitary (luteinizing hormone, follicle‐stimulating hormone, growth hormone) and thyroid hormones (thyroid stimulating hormone, T3, T4) were within normal ranges (Figure S3).^60^, ^70^, ^71^ ACTH transiently suppressed these hormones after 2 to 3 weeks;^60^, ^70^, ^71^ however, levels returned to baseline at treatment completion.^60^, ^70^, ^71^

##### Adrenal hormones (cortisol, intermediary steroids)

At IESS onset, only one study found blood cortisol was significantly higher in IESS compared with hospitalized controls.^72^ In studies without controls, blood cortisol was either in the normal range^66^, ^73^, ^74^, ^75^, ^76^ or elevated (Figure S3).^71^, ^72^, ^77^ CSF cortisol was lower in IESS than controls which reached significance in one study.^57^, ^67^, ^72^ Urinary cortisol was normal in two studies, examined without controls.^73^, ^74^

Eleven studies analysed the treatment effect of ACTH,^60^, ^65^, ^66^, ^70^, ^71^, ^72^, ^74^, ^76^, ^77^ hydrocortisone,^72^, ^77^, ^78^ and prednisone^75^ on cortisol. Most studies found ACTH/steroid treatment did not significantly alter blood cortisol levels from baseline (Figure S3).^60^, ^65^, ^66^, ^70^, ^71^, ^72^, ^74^, ^75^, ^76^, ^77^, ^78^ During the first 2 weeks of treatment, most studies found ACTH and hydrocortisone caused a transient increase in blood cortisol levels^60^, ^66^, ^70^, ^72^, ^74^, ^76^, ^77^ whereas prednisone suppressed levels.^65^ ACTH and hydrocortisone increased CSF cortisol in one study, yet no statistical analysis was provided.^72^ After 24 hours of ACTH, urinary cortisol increased from baseline^73^, ^74^ yet there was no control comparison or correlation with treatment response.^74^

Another hypothesis is that ACTH/steroids exert their antiseizure effect through ‘intermediary steroids’, such as the precursors of cortisol, aldosterone, and sex hormones. This was based on observations that progesterone and deoxycorticosterone prevented and reduced seizures in adults.^79^ No studies have compared intermediary steroid levels at IESS onset with controls. Testosterone precursors, dehydroepiandrosterone sulfate, and androstenedione are reportedly elevated or within normal range at baseline in IESS.^65^, ^73^, ^74^ ACTH significantly lowered dehydroepiandrosterone sulfate but not androstenendione,^65^ and higher baseline ratios of dehydroepiandrosterone sulfate:androstenedione correlated with ACTH response in some studies.^73^, ^74^ No correlations were found for cortisol or aldosterone precursors.^73^, ^74^ Other studies reported ACTH and hydrocortisone increased plasma/CSF 11‐hydroxycorticosteroid^78^ and general excretion of steroids;^80^ however, the findings lacked statistical comparison and have not been replicated.

#### Inflammation

We found 18 studies that examined possible neuroinflammation and immune dysregulation underlying IESS and the anti‐inflammatory effect of ACTH/steroids. Six studies quantified lymphocyte populations, five examined immunoglobulins, nine quantified cytokines/chemokines, one examined microglial activation, and three assessed integrity of the BBB.

##### Lymphocyte, T‐cell, and B‐cell population

**FIGURE 3:**
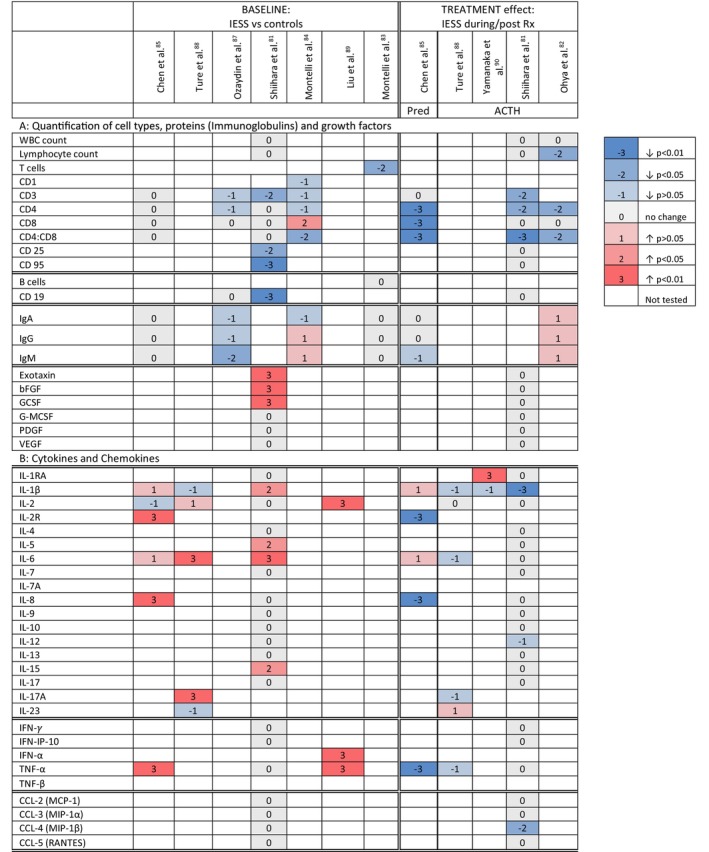
Inflammatory and immune profile in the blood of children with IESS at baseline and following treatment. Preliminary evidence that children with IESS have elevated pro‐inflammatory cytokines in blood compared with controls and may have altered T‐cell responses. ACTH and prednisone appear to suppress T‐cell‐mediated immunity yet do not consistently alter cytokine/chemokine levels. Variances were graded as follows: 0, no change; 1, elevated with *p* > 0.05; 2, elevated with *p* < 0.05; 3, elevated with *p* < 0.01; −1, reduced with *p* > 0.05; −2, reduced with *p* < 0.05; −3, reduced with *p* < 0.01; grading of CSF chemokine/cytokines in encephalitis based on Kothur et al.^153^ Abbreviations: ACTH, adrenocorticotrophin hormone; bFGF, basic fibroflast growth factor; CCL, chemokine ligand; CD, cluster of differentiation; GCSF, granulocyte colony‐stimulating factor; G‐MCSF, granulocyte‐macrophage colony‐stimulating factor; IESS, infantile epileptic spasms syndrome; IFN, interferon; Ig, immunoglobulin; IL, interleukin; PDGF, platelet‐derived growth factor; Pred, prednisone; Rx, treatment; TNF, tumour‐necrosis factor; VEGF, vascular endothelial growth factor; WBC, white blood cell count.

We have summarized the results from included studies examining immune cell types and cytokines/chemokines in the blood (Figure 3) and CSF (Figure S4) of children with IESS, compared with matched controls, and the treatment effect of ACTH and prednisone.

In a single study, lymphocyte count at IESS onset was no different to controls (Figure 3).^81^ ACTH significantly reduced lymphocytes in one study,^82^ whereas another found no change.^81^ Three of five studies found T‐cell subsets were significantly lower at IESS onset compared with controls.^81^, ^83^, ^84^, ^85^ After ACTH and prednisone, three studies reported reduced CD3, CD4, CD8 cell counts and CD4:8 ratio, consistent with suppression of cell‐mediated immunity.^81^, ^82^, ^85^ Helper T cells were more suppressed than cytotoxic T cells (Figure 3).^81^, ^82^, ^85^ Recovery of these T‐cell subsets was seen at 6 months after treatment.^82^ At IESS onset, B‐cell counts were either lower^81^ or no different to controls,^86^ and were unchanged by ACTH in a single study.^81^

Functional T‐cell studies were limited. At IESS onset, two studies reported reduced proliferation and migration of T cells after phytohaemagglutinin stimulation^83^, ^84^ although responses following ACTH/steroids were not performed. Dinitrochlorobenzene skin testing, which measures delayed hypersensitivity reactions, found that after ACTH the erythematous skin reaction was reduced or absent, indicating a significantly suppressed T‐cell response.^83^, ^84^

##### Immunoglobulins

Studies quantifying immunoglobulin‐A (IgA), IgG, IgM found no differences in children with IESS at baseline^83^, ^84^, ^85^, ^87^ and no changes after prednisone^85^ or ACTH treatment (Figure 3).^82^ At baseline, one study reported antibody formation against normal brain tissue, suggesting an abnormal immune response.^86^

##### Cytokines/chemokines

Four studies analysed the cytokine/chemokine profile in serum (Figure 3).^81^, ^85^, ^88^, ^89^ At IESS onset, children had significantly higher serum levels of the following pro‐inflammatory cytokines: interleukin‐1β (IL‐1β), IL‐2R, IL‐6, IL‐8, and IL‐15, IL‐17A, tumour‐necrosis factor‐α (TNF‐α), and interferon‐α^81^, ^85^, ^88^, ^89^ whereas the anti‐inflammatory cytokines IL‐1RA, IL‐4, and IL‐10 were not significantly different (Figure 3).^81^ Most studies found IL‐1β was unaltered by prednisone/ACTH;^85^, ^88^, ^90^ however, one reported a significant reduction.^81^ Prednisone,^85^ but not ACTH, reduced IL‐2R, IL‐8, and TNF‐α; however, IL‐2 and IL‐6 were unchanged.^81^, ^85^, ^88^ A single study found chemokines did not differ from controls, and were not changed by ACTH.^81^

Two studies^57^, ^91^ examined the cytokine/chemokine profile in CSF (Figure S4). CSF IL‐1RA was significantly lower in IESS than controls,^57^, ^91^ which was not observed in blood.^81^ One study found detectable or elevated IL‐1β levels in IESS with a known aetiology (compared with unknown).^92^ Two infants successfully treated with ACTH had repeat CSF testing; IL‐1β normalized in one and remained detectable in the other.^92^ One study investigated CSF neopterin, finding one of four infants had elevated levels; however, treatment effect was not analysed.^93^

##### Microglial activation

One study^94^ examined for microglial activation in children with unknown IESS aetiology using positron emission tomography scans and a tracer that binds to a translocator protein which is expressed by activated microglia. Five of eight children had focal areas of increased binding, suggesting neuroinflammation, which reduced or normalized following ACTH.^94^ The significance of these findings was limited by the lack of control comparison and lack of replication.

##### BBB function

Three studies measured CSF and serum protein and albumin levels as indirect evidence of BBB damage, with conflicting results.^63^, ^95^, ^96^ Two studies found no evidence of BBB damage, as CSF albumin and CSF:serum albumin ratios were normal and were unaltered following ACTH.^63^, ^95^ One study reported elevated CSF protein/albumin which normalized after dexamethasone and ACTH.^96^ Findings were limited by the lack of comparison with typically developing controls and by the presumption that CSF protein/albumin levels reliably reflect BBB damage.

One study analysed levels of serum matrix metallopeptidase‐9 (MMP‐9) as a marker of BBB damage. MMP‐9 is an enzyme in the cerebral endothelium, which degrades collagen and proteins of the BBB.^95^ MMP‐9 levels did not differ between aetiological subgroups of IESS. ACTH responders had significantly higher MMP‐9 levels, and a higher ratio of MMP‐9 to its inhibitor (TIMP‐1). The authors inferred ACTH may restore BBB integrity, yet their findings were limited by the lack of control comparison and serial measurements. Another study measured CSF:serum IgG index as evidence of a CNS immune response; however, it was within normal ranges before and during ACTH treatment.^63^

#### Neural transmission and connectivity

Twenty‐two studies explored the hypothesis that IESS results from altered neural transmission and connectivity and that ACTH/steroids may modify signalling pathways or neuronal function (Figure S1). Twelve studies examined neurotransmitters and excitatory or inhibitory amino acids, seven studies analysed neural pathways, and four examined markers of neuronal health as a measure of neuronal excitability.

##### Neurotransmitters and amino acids

**FIGURE 4:**
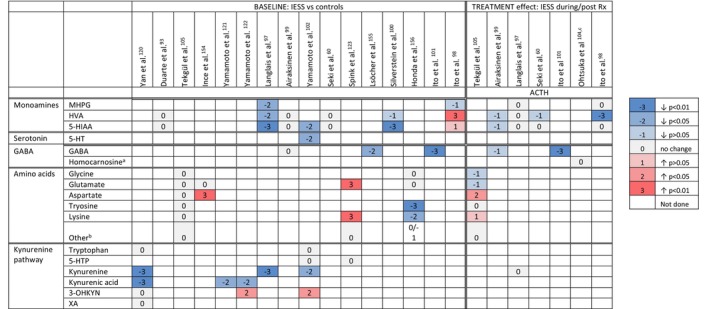
Metabolite profile of monoamines, amino acids, and the kynurenine pathway in the cerebrospinal fluid of children with IESS at baseline and following treatment. Evidence indicating CSF GABA and kynurenine pathway metabolites are reduced in children with IESS at epileptic spasm onset without a clear or consistent response to ACTH treatment. ^a^An inhibitory neurotransmitter produced from GABA and histidine. ^b^Phenylalanine, methionine, threonine, proline, arginine, isoleucine, leucine, alanine, tyrosine, serine, hydroxyproline, cysteine, valine, ornithine, glutamine, histidine, taurine. ^c^Baseline higher than reference range (no control comparison). Abbreviations: 3‐OHKYN, 3‐hydroxykynurenine; 5‐HIAA, 5 hydroxyindoleacetic acid; 5‐HT, serotonin; 5‐HTP, 5‐hydroxytryptophan; ACTH, adrenocorticotrophin hormone; GABA, γ‐aminobutyric acid; HVA, homovanillic acid; IESS, infantile epileptic spasms syndrome; MHPG, 3‐methoxy‐4‐hydroxyphenylglycol; XA, xanthurenic acid.

Several studies investigated whether IESS is associated with disturbances of primary monoamines in the CSF: noradrenaline,^97^, ^98^ dopamine,^60^, ^93^, ^97^, ^98^, ^99^, ^100^, ^101^ and serotonin.^60^, ^93^, ^97^, ^98^, ^99^, ^100^, ^102^ Most studies found metabolites in IESS were either lower^97^, ^100^, ^102^ or no different^60^, ^93^, ^99^ to controls (Figure 4). Only one study found dopamine metabolites were elevated.^98^ ACTH treatment did not significantly alter primary monoamines.^60^, ^97^, ^98^, ^99^

Studies examining whether an imbalance of CSF inhibitory (γ‐aminobutyric acid [GABA], glycine) or excitatory (aspartate, glutamate) amino acids may lead to IESS have produced variable results (Figure 4). In two of three studies, CSF GABA was lower in IESS than controls^101^, ^103^ and significantly lower in IESS with a known aetiology.^99^ ACTH reduced CSF GABA, reaching significance in one of two studies.^99^, ^101^ ACTH also reduced homocarnosine, an inhibitory hormone produced from GABA and histidine^104^ yet these findings were limited by the lack statistical analyses and comparison with controls. One study found ACTH significantly increased aspartate;^105^ however, other amino acids were unchanged.^105^ Magnetic resonance spectroscopy found no difference in glutamine/glutamate metabolites in IESS compared with controls at baseline.^106^ ACTH treatment lowered the glutamine/glutamate signal; however, this normalized on repeat imaging 6 to 11 months later.^106^

##### Neural pathways and connectivity

Evidence supporting altered neuronal pathways in IESS was based on early autopsy findings that children with IESS and intellectual impairment had reduced dendritic branching and growth of cortical neurons compared with controls.^107^, ^108^ One group proposed that IESS originates from brainstem dysfunction and altered serotonergic pathways after identifying common brainstem abnormalities and altered histological expression of neurotransmitters.^109^ Subsequent larger autopsy case series have disputed these brainstem abnormalities.^110^ Three studies used functional MRI (fMRI) analyses in IESS to assess default motor networks or connectivity between brain regions. One study investigated neuronal networks that were activated with EEG changes in IESS compared with controls with focal seizures. In IESS, cortical areas were activated in regions with EEG activity and activation was also seen in the deep grey nuclei and occipital regions correlating with EEG slowing.^111^ Two studies described the same IESS cohort compared with non‐seizure controls and found infants with IESS had disrupted connectivity between regions of the brain that should be well connected in a resting state, the so‐called default mode network.^112^, ^113^ None of the studies examined the effect of ACTH/steroids on neuronal connectivity. One study found improvement of previously disrupted inter‐regional brain connectivity after epilepsy surgery; however, specific data for the two IESS infants were not available.^114^

##### Markers of neuronal health

Neuronal cell health was analysed by measuring four CSF markers of neuronal injury. Prolactin and nitric oxide metabolites are seen as markers of excitotoxicity. Tau protein is an axonal microtubule‐associated protein and neuron‐specific enolase is an enzyme within neurons released by damaged cells.^60^, ^115^ All four markers, prolactin,^116^ tau protein,^115^ neuron‐specific enolase,^60^ and nitric oxide metabolites,^117^ were higher in IESS than controls and rose significantly during ACTH treatment. This is indirect evidence of neuronal cell injury/dysfunction in IESS. The authors hypothesized that ACTH may promote death of damaged neurons followed by neuronal repair or regeneration. However, this is speculative; it could also imply that ACTH does not alter the natural history of IESS.

#### Metabolic pathways

Thirty‐three studies explored whether altered metabolic pathways and/or cellular stress contribute to IESS onset and whether ACTH/steroids modify these processes (Figure S1). Nine studies examined the kynurenine pathway, 19 investigated neuronal cell and cerebral metabolism, and five examined growth factors that may regulate neuronal cell survival.

##### Kynurenine pathway

Early studies identified that ACTH, prednisone, and dexamethasone normalized the urinary excretion of metabolites following a tryptophan load, providing the first evidence that ACTH/steroids alter the kynurenine (KYN) pathway.^118^, ^119^ Several studies have since confirmed that children with IESS have significantly lower CSF levels of neuroprotective metabolites KYN^97^, ^102^, ^120^ and kynurenic acid (KYNA),^120^, ^121^, ^122^ and a lower KYNA/KYN ratio^120^ than controls. CSF 3‐hydroxykynurenine (3‐OHKY), a neurotoxic subsidiary of KYN, is significantly elevated^102^, ^122^ whereas tryptophan did not differ.^102^, ^123^ One study analysed ACTH treatment, which did not alter CSF KYN level,^97^ while another noted that responders to prednisone had lower baseline CSF KYN and KYNA/KYN than partial or non‐responders.^120^

One case series analysed stool microbiota and serum metabolites of the KYN pathway to determine whether microbiome–metabolome interactions affected ACTH treatment response.^124^ Before ACTH, non‐responders had larger populations of *Clostridioides* and *Peptoclostridium*. This bacterial upregulation correlated with the upregulation of xanthurenic acid. This implied that ACTH treatment failure may result from increased gut microbiota disturbing KYN metabolism.

##### Neuronal cell and cerebral metabolism

Neuronal cell metabolism was analysed by serum and CSF markers and various imaging modalities. Serum and CSF markers indicating cellular stress, including lactate, pyruvate, and lactate dehydrogenase, were normal at IESS onset.^125^, ^126^ Following ACTH, lactate and pyruvate significantly increased.^125^

Using magnetic resonance spectroscopy, *N*‐acetylaspartate, a metabolite produced by neurons/axons, was no different to controls at IESS onset,^106^ and ACTH significantly lowered the *N*‐acetylaspartate peak, suggesting reduced neuronal activity/energy metabolism.^106^, ^127^ Choline peaks, which reflect cell membrane synthesis and degradation, were unaltered.^106^, ^127^ Phosphorus metabolites were no different to controls at baseline and were unaltered by ACTH, suggesting oxidative metabolism was not affected.^128^ ACTH reduced the ratio of myelin and phospholipid precursors, which is observed as the brain matures, indicating ACTH may alter phospholipid metabolism and myelination, although this is speculative.^128^

Positron emission tomography and single photon‐emission computed tomography studies have identified both focal and diffuse areas of cortical and subcortical hypo‐ and hypermetabolism.^129^, ^130^, ^131^, ^132^, ^133^, ^134^, ^135^ Only one study compared IESS to controls and did not identify a consistent anatomical focus.^136^ Focal imaging changes correlated with focal EEG abnormalities^129^, ^132^ and generally resolved on follow‐up scans confirming their transient nature (apart from cases of cortical dysplasia).^129^, ^131^, ^132^ Single photon‐emission computed tomography and carotid ultrasound studies were performed to determine whether ACTH/steroids alter cerebral blood flow and metabolism.^137^, ^138^, ^139^, ^140^, ^141^, ^142^ Only one study compared IESS with controls, reporting increased cerebral blood flow compared with typically developing children, yet no difference to epilepsy controls.^142^ ACTH and hydrocortisone reduced cerebral blood flow; however, it remained within normal ranges^142^ or no statistical analyses were provided.^137^, ^138^, ^139^, ^140^, ^141^, ^142^

##### Growth factors

β‐Nerve growth factor (β‐NGF) and insulin‐like growth factor 1 (IGF‐1) regulate neuronal cell growth/survival and early brain development. CSF β‐NGF and IGF‐1 did not differ in IESS with unknown aetiology from controls yet were significantly lower in IESS with a known cause such as trisomy 21 or stroke.^68^, ^143^, ^144^ Histological studies also confirmed loss of IGF‐1 cortical neurons in children with IESS due to stroke.^145^ ACTH response correlated with higher β‐NGF and IGF‐1 at baseline;^68^, ^144^ however, pre–post‐treatment response lacked statistical comparisons.^144^

One study quantified growth factors and found elevated exotoxin, basic fibroblast growth factor, and granulocyte colony‐stimulating factor in IESS compared with controls (Figure 3); however, no changes were detected following ACTH.^81^ The hypothesis that ACTH enhances the action of growth factors to rescue neurons from apoptosis and improves neuronal or synaptic connections remains speculative.

## DISCUSSION

The body of clinical research over the past 65 years summarized here reflects five key hypotheses about the aetiology of IESS and how ACTH/corticosteroids may exert their effect. They include altered gene regulation, stress, neuroinflammation, altered neuronal networks, and altered metabolic pathways. It is likely that these mechanisms interact and coexist, rather than function independently, and supports the concept that IESS is a complex neurodevelopmental disorder. For example, it is described that early life stressors, trauma, and/or inflammation may cause epigenetic modifications which alter gene expression^146^ and may increase expression of vulnerability genes in IESS. Given recent advances in bioinformatic analysis, it may be that multi‐omics analyses could provide further insights into how these pathways interact or differ in IESS compared with controls or between aetiological subgroups at the DNA, RNA, protein, and metabolite levels. More integrated and standardized approaches may advance or refine some of the earlier hypotheses, for example by investigating the effects of stress through epigenetic analyses rather than the quantification of HPA‐axis hormones.

The current evidence supporting altered gene and epigenetic regulation in IESS is limited. Only 23% to 25% of IESS cases^17^, ^19^, ^29^, ^30^, ^32^ are explained by monogenic highly penetrant variants which is lower than the 30% to 35% frequency in developmental and epileptic encephalopathies.^22^, ^27^ The GO pathways identified in IESS in our review and previous studies encompassed themes of brain development, epigenetic control of cellular processes, and neurotransmission. While cellular transport was a common theme in IESS and other infantile‐onset epilepsies, GO pathways in the latter group differed in that they involved motor activity pathways such as cell motility and ion transport and cellular behavioural responses due to stimuli.^42^ The heterogeneity in biological pathways indicates that the therapeutic effect of ACTH/steroids is plausible at a more regulatory rather than specific gene/protein level. Given 10% to 20% of the genome is directly regulated by glucocorticoids,^147^ it is plausible that ACTH/steroids directly alter gene expression by activating or repressing transcription/translation^148^, ^149^ or modify gene expression by indirect mechanisms such as altering transcription factors, RNA (mRNA, non‐coding RNA), or epigenetic modifications.^148^, ^149^ However, no studies have definitively demonstrated this in IESS.

Evidence supporting HPA‐axis activation or ‘CRH excess’ at IESS onset remains largely speculative. Although one study demonstrated increased expression of CRH and CRHR1 in the brain tissue of infants with IESS, this was not a clear baseline comparison as infants were undergoing surgery for refractive epilepsy and had been treated with numerous medications.^56^ Five studies have reliably identified a CSF ACTH deficiency in IESS compared with controls; however, treatment fails to correct this deficiency. The few studies examining other HPA‐axis hormones have failed to identify direct evidence of HPA‐axis activation or clear changes following treatment. The methodological limitations of the current evidence make it difficult to definitively exclude the hypothesis that ACTH/steroids alter the HPA‐axis hormones, although it seems unlikely that this mechanism alone controls epileptic spasms. Most studies have been limited by small sample sizes, the lack of comparison with a typically developing control group, failure to repeat baseline measures, or inadequate method of assessment and analysis.

The literature supporting neuroinflammation in IESS is limited. Four studies consistently found elevated serum cytokines compared with controls; however, CSF cytokine studies, measures of BBB integrity, and functional imaging studies were either normal or lacked controls or replication studies. Most studies have examined blood or CSF which are less specific than brain tissue and may not reflect processes within the CNS. There is some evidence suggesting ACTH/steroids may suppress T‐cell‐mediated responses and certain cytokines; however, these studies have not been replicated, and it is not clear whether this is truly altering the disease process in IESS or is an expected effect of ACTH/steroids. The lack of direct evidence supporting neuroinflammation makes it difficult to draw definitive conclusions. We acknowledge the inherent difficulties accessing brain tissue in this population and even future use of CSF biomarkers may prove challenging, as in our experience infants with clear structural aetiologies do not routinely have CSF collected and access to post‐treatment CSF samples may be limited by ethical research practices.

The current evidence examining neural transmission in IESS suggests there are no significant measurable and consistent disturbances in monoamine, excitatory, or inhibitory neurotransmission in IESS. CSF GABA was lower in IESS than controls in two studies, yet there were no consistent changes following treatment, even though vigabatrin is known to have efficacy in TSC and non‐TSC related IESS. The quality of these studies is limited by similar factors described in the literature on the HPA‐axis, including small samples, lack of control comparison, and lack of replication studies. There is minimal evidence supporting abnormalities in neuronal cell function. The evidence is largely derived from early autopsy studies or indirect measures of neuronal cell health. Studies interpreting brain connectivity are limited by the absence of normative fMRI data in the developing brain. Future studies examining neural pathways and connectivity in IESS may be strengthened by analysing a combination of neuroimaging modalities including fMRI or tractography accompanied by non‐invasive testing such as EEG or, if feasible, transcranial stimulation.

Lastly, evidence examining the KYN pathway in IESS identified consistent findings that CSF KYN, KYNA, and the KYNA:KYN ratio were lower in IESS than controls and predicted response to steroid treatment in a single study. At a biological level, KYN crosses the BBB and is either converted to KYNA in astrocytes or metabolized in the microglia to quinolinic acid. These metabolites are known to be involved in epilepsy and affect synaptic transmission, inflammation, and immune responses.^150^, ^151^ KYNA is a glutamate (NMDA) receptor antagonist and has anti‐inflammatory properties, whereas quinolinic acid stimulates the NMDA receptor and may be responsible for excitatory neuronal damage.^150^ Further longitudinal studies may confirm the utility of CSF KYN metabolites as a biomarker of treatment response, albeit in a limited population of infants with non‐structural pathologies. Evidence of cellular stress and cerebral/neuronal cell dysfunction is inconsistent and assumptions that ACTH may regulate cell survival and neurogenesis remain hypothetical.

The current evidence examining the effect of ACTH/steroids in children with IESS is limited and largely hypothetical. Findings have been inconsistent, which may reflect the heterogeneity in IESS aetiology and treatment response. Reliable and definitive conclusions have been limited by the methodological heterogeneity of studies and the lack of replication of findings. The quality of most studies is limited by small sample sizes, the lack of comparison with a typically developing control group, failure to repeat baseline measures, or inadequate method of assessment and analysis. Most evidence is indirect as there are inherent difficulties accessing brain tissue in this population. As a result, many studies used blood and CSF which are less specific than brain tissue and may not reflect processes within the central nervous system.

It is recommended that future prospective studies use multi‐omics sequencing to compare large IESS cohorts with controls to explore gene regulation and the effects of stress/injury/inflammation. Bulk and single‐cell approaches including transcriptomics, proteomics, and metabolomics may highlight differences between individuals and aetiological groups at a biological level to explain the heterogeneity of IESS and treatment response. Longitudinal data collection, as evidenced by the EPISTOP multi‐omics analysis in TSC,^152^ may clearly identify biomarkers of disease progression or treatment response. This may enable individualized treatment protocols in IESS based on the biological mechanisms identified, rather than the current ‘one‐size‐fits‐all’ approach. Longitudinal studies examining ACTH, steroids, and vigabatrin will better inform us of the long‐term neurodevelopmental outcomes or potential harms of treatment. To improve comparability across studies, from the past and future, it is recommended that studies adopt standardized protocols for the measurement of biomarkers and collection of clinical data such as EEG and imaging.

## CONCLUSION

The current evidence examining the aetiology and effect of ACTH/steroids in children with IESS is limited and largely hypothetical. Findings have been inconsistent, which may reflect both the heterogeneity in IESS aetiology and treatment response as well as the methodological heterogeneity of studies and lack of reproducibility. Large prospective IESS cohorts with appropriately matched controls will be required to determine differences and commonalities between IESS with a known compared with unknown aetiology and whether ACTH/steroids alter common pathways to control epileptic spasms. An improved understanding of the pathophysiology of IESS and the mechanism of action of ACTH/corticosteroids may lead to new disease‐modifying treatments to improve long‐term neurodevelopment outcomes.

## FUNDING INFORMATION

EAI received stipend support to complete this work from NHMRC investigator grant APP1193648, Petre Foundation.

## CONFLICT OF INTEREST STATEMENT

The authors have stated that they had no interests that might be perceived as posing a conflict or bias.

## Supporting information


**Appendix S1:** Methodology for review.

**Appendix S2:** Methodology for assessing genetic disorders in IESS.

**Figure S1:** Flow diagram of studies selected for inclusion in review.

**Figure S2:** Hormonal profile of the HPA‐axis in the CSF of children with IESS at baseline and following treatment.

**Figure S3:** Hormonal profile of the HPA‐axis in the blood of children with IESS at baseline and following treatment.

**Figure S4:** Inflammatory and immune profile in the CSF children with IESS at baseline and following treatment.

## Data Availability

The data that support the findings of this study are available from the corresponding author upon reasonable request.
