# Supplementary material for: Aetiopathogenesis of infantile epileptic spasms syndrome and mechanisms of action of adrenocorticotrophin hormone/corticosteroids in children: A scoping review
Source: Dev Med Child Neurol. 2025 Feb 28;67(8):1004–25. doi: 10.1111/dmcn.16273 (PMC12237231; doi:10.1111/dmcn.16273)
Supplement: Supplementary file 1 — Appendix S1: Methodology for review. [file DMCN-67-1004-s004.docx]

**Appendix S1: Methodology for review**

The methodological workflow and process of selecting studies for inclusion is presented in Supplementary Figure 1. Exemplar PubMED search strategy below with limits applied.

| Search History | |
| --- | --- |
| 1 | Infantile spasms |
| 2 | infantile epileptic spasm syndrome.mp |
| 3 | spasms |
| 4 | epileptic spasms.mp |
| 5 | west syndrome |
| 6 | 1 or 2 or 3 or 4 or 5 or 6 |
| 7 | steroids |
| 8 | corticosteroids.mp |
| 9 | prednisone |
| 10 | hydrocortisone |
| 11 | dexamethasone |
| 12 | methylprednisolone |
| 13 | adrenocorticotrophin hormone |
| 14 | ACTH.mp |
| 15 | Tetracosactide.mp |
| 16 | treatment |
| 17 | 7 or 8 or 9 or 10 or 11 or 12 or 12 or 14 or 15 or 16 |
| 18 | biomarkers |
| 19 | Effect.mp |
| 20 | Action.mp |
| 21 | Change.mp |
| 22 | mechanism |
| 23 | 18 or 19 or 20 or 21 or 22 or 23 or 24 |
| 24 | 6 AND 17 AND 23 |
| 25 | Limit 24 to (humans and “all child (0 to 18 years)” and full-text article) |
| 26 | aetiology |
| 27 | cause |
| 28 | Gene.mp |
| 29 | Genetic |
| 30 | metabolic |
| 31 | inflammation |
| 32 | immune |
| 33 | excitation |
| 34 | neurotransmitters |
| 35 | 26 or 27 or 28 or 29 or 30 or 31 or 32 or 33 or 34 |
| 36 | 6 AND 23 AND 35 |
| 37 | Limit 24 to (humans and “all child (0 to 18 years)” and full-text article) |

After initial search and three consensus meetings to finalise hypotheses (supplementary figure 1), keywords based on each hypothesis were used to search the same databases and a similar filtering process for inclusion/exclusion was applied to these search results. For example, Kynurenine was used as key word and a title term combined with “AND” (Infantile spasms OR infantile epileptic spasm syndrome.mp OR IESS). The last search was conducted on 20^th^ December 2023.
