# Supplementary material for: Aetiopathogenesis of infantile epileptic spasms syndrome and mechanisms of action of adrenocorticotrophin hormone/corticosteroids in children: A scoping review
Source: Dev Med Child Neurol. 2025 Feb 28;67(8):1004–25. doi: 10.1111/dmcn.16273 (PMC12237231; doi:10.1111/dmcn.16273)
Supplement: Supplementary file 2 — Appendix S2: Methodology for assessing genetic disorders in IESS. [file DMCN-67-1004-s001.docx]

**Appendix S2: Methodology for assessing genetic disorders in IESS**

Three online databases, PubMED, MEDLINE and EMBASE databases were searched from 2010-December 2023 for IESS cohort studies examining genetic aetiology and the yield of genetic testing. Studies included met the following criteria: (1) a clearly defined IESS cohort with 2) individual data confirming the genetic diagnosis (obtained from the main publication or supplementary data), (3) IESS cohort of ≥ 5 participants, (4) sufficient data to confirm and categorise aetiological groups according to ILAE guidelines (structural, genetic, infectious, metabolic, immune and unknown). To present the genetic aetiology clearly we further divided the structural group into structural (acquired/congenital) causes such as brain injury, stroke and genetic-structural disorders such as TSC and disorders of neuronal migration. Studies were excluded that (1) described IESS cohorts associated with a single gene, (2) generalised results across other cohorts such as IESS and Lennox-Gaustaut Syndrome without providing individual IESS data or (3) were written in languages other than English.

Search terms included the following keywords and derivatives: “infantile epileptic spasm syndrome”, “infantile spasms”, “spasms”, “epileptic spasms”, “West syndrome”, “aetiology”, “cause”, “classification”, “diagnosis”, “gene”, “genetic”, “genotype”, “genomic”, “test”, “yield”, “sequencing”, “exome”. References were also hand-searched.

After screening titles and abstracts, 32 studies were identified that met inclusion criteria. Two studies included previously published cohorts (1, 2)^[[1]](#footnote-1)^ as such the most recent publication was included. After reading full-text articles, 6 studies were excluded as they had insufficient data to categorise aetiological groups/confirm genetic diagnosis, included less than five IESS participants or were not written in English. (3-8)

1. 1. Peng J, Wang Y, He F, Chen C, Wu LW, Yang LF, et al. Novel West syndrome candidate genes in a Chinese cohort. CNS Neurosci Ther. 2018;24(12):1196-206.

   2. Allen AS, Berkovic SF, Cossette P, Delanty N, Dlugos D, Eichler EE, et al. De novo mutations in epileptic encephalopathies. Nature. 2013;501(7466):217-21.

   3. Chourasia N, Yuskaitis CJ, Libenson MH, Bergin AM, Liu S, Zhang B, et al. Infantile spasms: Assessing the diagnostic yield of an institutional guideline and the impact of etiology on long-term treatment response. Epilepsia. 2022;63(5):1164-76.

   4. Hu C, Liu D, Luo T, Wang Y, Liu Z. Phenotypic spectrum and long‐term outcome of children with genetic early‐infantile‐onset developmental and epileptic encephalopathy. Epileptic disorders. 2022;24(2):343-52.

   5. Mitta N, Menon RN, McTague A, Radhakrishnan A, Sundaram S, Cherian A, et al. Genotype-phenotype correlates of infantile-onset developmental & epileptic encephalopathy syndromes in South India: A single centre experience. Epilepsy Res. 2020;166:106398.

   6. Mercimek-Mahmutoglu S, Patel J, Cordeiro D, Hewson S, Callen D, Donner EJ, et al. Diagnostic yield of genetic testing in epileptic encephalopathy in childhood. Epilepsia. 2015;56(5):707-16.

   7. Juanes M, Loos M, Reyes G, Veneruzzo G, García FM, Aschettino G, et al. Clinical and genetic study of developmental and epileptic encephalopathy in Argentinean pediatric patients. Medicina (B Aires). 2022;82(6):856-65.

   8. Duc NM, Thu NTM, Bui CB, Hoa G, Le Trung Hieu N. Genotype and phenotype characteristics of West syndrome in 20 Vietnamese children: Two novel variants detected by next-generation sequencing. Epilepsy Res. 2023;190:107094. [↑](#footnote-ref-1)
