# Supplementary figures and images for: Aetiopathogenesis of infantile epileptic spasms syndrome and mechanisms of action of adrenocorticotrophin hormone/corticosteroids in children: A scoping review
Source: Dev Med Child Neurol. 2025 Feb 28;67(8):1004–25. doi: 10.1111/dmcn.16273 (PMC12237231; doi:10.1111/dmcn.16273)

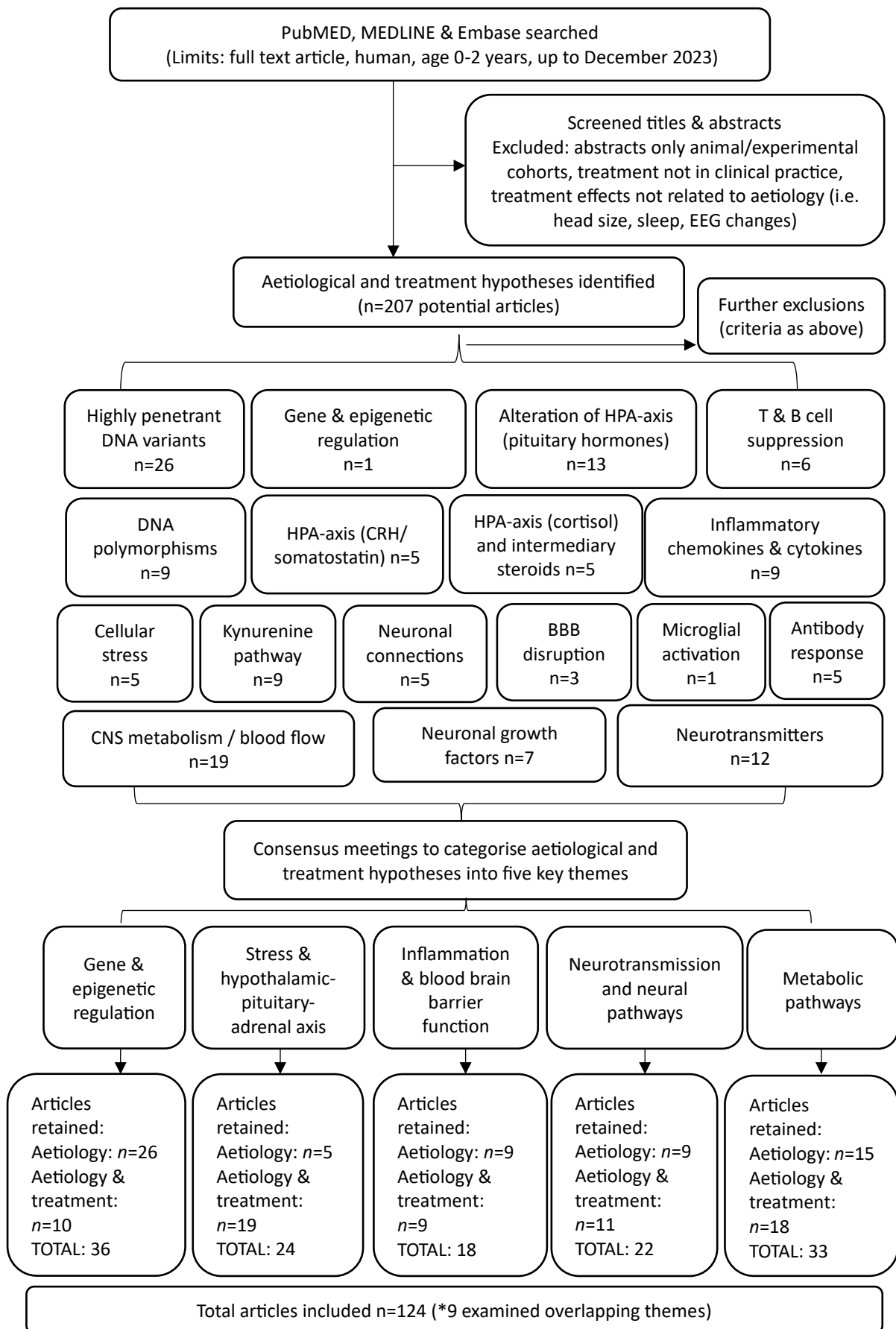

Supplement: Supplementary file 3 — Figure S1: Flow diagram of studies selected for inclusion in review. [file DMCN-67-1004-s005.pdf]
