# Supplementary material for: Aetiopathogenesis of infantile epileptic spasms syndrome and mechanisms of action of adrenocorticotrophin hormone/corticosteroids in children: A scoping review
Source: Dev Med Child Neurol. 2025 Feb 28;67(8):1004–25. doi: 10.1111/dmcn.16273 (PMC12237231; doi:10.1111/dmcn.16273)
Supplement: Supplementary file 4 — Figure S2: Hormonal profile of the HPA‐axis in the CSF of children with IESS at baseline and following treatment. [file DMCN-67-1004-s006.docx]

**Supplementary Figure 2: Hormonal profile of the HPA-axis in the CSF of children with IESS at baseline and following treatment**

|  |  | BASELINE: IESS vs controls | | | | | | | | | TREATMENT effect: IESS during/post Rx | | | | | | | | | | |
| --- | --- | --- | --- | --- | --- | --- | --- | --- | --- | --- | --- | --- | --- | --- | --- | --- | --- | --- | --- | --- | --- |
|  |  | Aydln et al 2002 | Nagamitsu et al 2001 | Hirai et al 1998 | Baram et al 1995 | Baram et al 1992 | Seki et al 1990 | Nalin et al 1985 | Facchinetti et al 1985 | Hagishima et al 1972a | Aydln et al 2002 | Hirai et al 1998* | Heiskala et al 1997 | Seki et al 1990* | Riikonen et al 1988 | Nalin et al 1985 | Facchinetti et al 1985 | Hagishima et al 1972a | Fukazawa et al 1972 | Hagishima et al 1972a | Fukazawa et al 1972 |
|  |  |  |  |  |  |  |  |  |  |  | ACTH | | | | | | | | | Hydrocortisone | |
| Hypothalamic | CRH |  | -1 |  |  | 0 |  |  |  |  |  |  |  |  |  |  |  |  |  |  |  |
|  | Somatostatin |  |  | 2 |  |  | -1 |  |  |  |  | -1 |  | -1 |  |  |  |  |  |  |  |
| Pituitary | ACTH |  | -3 |  | -3 | -2 |  | -2 | -2 |  |  |  | -1 |  | -1 | 1 | 1 |  |  |  |  |
|  | β-endorphin |  | -3 |  |  |  |  | 0 | 0 |  |  |  | -1 |  |  | 0 | 0 |  |  |  |  |
|  | Prolactin | 3 |  |  |  |  |  |  |  |  | 2 |  |  |  |  |  |  |  |  |  |  |
| Adrenal | Cortisol |  |  |  | -3 | -1 |  |  |  | 1 |  |  |  |  |  |  |  | 1 |  | 1 |  |
|  | 11-OHCS |  |  |  |  |  |  |  |  |  |  |  |  |  |  |  |  |  | 1 |  | 1 |

| -3 | ↓ p<0.01 |
| --- | --- |
| -2 | ↓ p<0.05 |
| -1 | ↓ p>0.05 |
| 0 | no change |
| 1 | ↑ p>0.05 |
| 2 | ↑ p<0.05 |
| 3 | ↑ p<0.01 |
|  | Not done |

Key: CRH=corticotrophin releasing hormone, ACTH=adrenocorticotrophin hormone, 11-OHCS=11-hydroxycorticosteroid, *pyridoxal phosphate given prior to ACTH
