# Supplementary material for: Aetiopathogenesis of infantile epileptic spasms syndrome and mechanisms of action of adrenocorticotrophin hormone/corticosteroids in children: A scoping review
Source: Dev Med Child Neurol. 2025 Feb 28;67(8):1004–25. doi: 10.1111/dmcn.16273 (PMC12237231; doi:10.1111/dmcn.16273)
Supplement: Supplementary file 5 — Figure S3: Hormonal profile of the HPA‐axis in the blood of children with IESS at baseline and following treatment. [file DMCN-67-1004-s002.pdf]

Supplementary Figure 3: Hormonal profile of the HPA-axis in the blood of children with IESS at baseline and following treatment

		BASELINE: IESS vs controls		TREATMENT effect: IESS during/post Rx												
		Nalin et al 1985	Hagishima et al 1972a	Yamamoto et al 1998	Kusse et al 1993	Seki et al 1990*	Snead et al 1989	Riikonen et al 1986	Izumi et al 1984/85	Sugie 1983b	Hagishima et al 1972a/b	Fukazawa et al 1972	Yamamoto et al 1998	Hagishima et al 1972a/b	Fukazawa et al 1972	Farwell et al 1984
				ACTH									Dexamethasone	Hydrocortisone		Prednisone
Hypothalamic	CRH															
	Somatostatin															
Pituitary	ACTH	0			1		1									
	β-endorphin	0														
	LH			0		0							0			
	FSH			0		0							0			
	GH			0		0			0				0			
	TSH			0		0	0						0			
	Prolactin					0	0									
Adrenal	Cortisol		3	0	-1	0	1	1	1	1	1		0	1		-1
	DHEAS				-2			0								
	Androstenedione				0											
	11-OHCS											1			1	
Thyroid	T3, T4					0	0		0				0			

-3	↓ $p<0.01$
-2	↓ $p<0.05$
-1	↓ $p>0.05$
0	no change
1	↑ $p>0.05$
2	↑ $p<0.05$
3	↑ $p<0.01$
	Not done

Key: CRH=corticotrophin releasing hormone, ACTH=adrenocorticotrophin hormone, LH=Luteinising hormone, FSH=Follicle-stimulating hormone, GH=growth hormone, TSH=thyroid stimulating hormone, DHEAS=dehydroepiandrosterone sulfate, 17-OHP=17-hydroxyprogesterone, 11-OHCS=11-hydroxycorticosteroid, T3=triiodothyronine, T4=thyroxine, *pyridoxal phosphate given prior to ACTH
