# Supplementary material for: Aetiopathogenesis of infantile epileptic spasms syndrome and mechanisms of action of adrenocorticotrophin hormone/corticosteroids in children: A scoping review
Source: Dev Med Child Neurol. 2025 Feb 28;67(8):1004–25. doi: 10.1111/dmcn.16273 (PMC12237231; doi:10.1111/dmcn.16273)
Supplement: Supplementary file 6 — Figure S4: Inflammatory and immune profile in the CSF children with IESS at baseline and following treatment. [file DMCN-67-1004-s003.docx]

**Supplementary Figure 4: Inflammatory and immune profile in the CSF children with IESS at baseline and following treatment**

|  | BASELINE:  IESS vs controls | | TREATMENT effect: IESS during/post Rx |  |
| --- | --- | --- | --- | --- |
|  | Haginoya et al 2009 | Baram et al 1992 | Sousa et al 2012 |  |
|  |  |  | ACTH |  |
| IL-1RA | -2 |  |  |  |
| IL-1β | -1 | -1 | -1 |  |
| IL-2 |  |  |  |  |
| IL-2R |  |  |  |  |
| IL-4 |  |  |  |  |
| IL-5 |  |  |  |  |
| IL-6 | -1 |  |  |  |
| IL-7 |  |  |  |  |
| IL-7A |  |  |  |  |
| IL-8 |  |  |  |  |
| IL-9 |  |  |  |  |
| IL-10 |  |  |  |  |
| IL-12 |  |  |  |  |
| IL-13 |  |  |  |  |
| IL-15 |  |  |  |  |
| IL-17 |  |  |  |  |
| IL-17A |  |  |  |  |
| IL-23 |  |  |  |  |
| IFN-𝛾 |  |  |  |  |
| IFN-IP-10 |  |  |  |  |
| IFN-α |  |  |  |  |
| TNF-α | -1 |  |  |  |
| TNF-β |  |  |  |  |

| -3 | ↓ p<0.01 |
| --- | --- |
| -2 | ↓ p<0.05 |
| -1 | ↓ p>0.05 |
| 0 | no change |
| 1 | ↑ p>0.05 |
| 2 | ↑ p<0.05 |
| 3 | ↑ p<0.01 |
|  | Not done |
